# Influence of Sorghum Kafirin on Serum Lipid Profile and Antioxidant Activity in Hyperlipidemic Rats (*In Vitro* and *In Vivo* Studies)

**DOI:** 10.1155/2015/164725

**Published:** 2015-10-26

**Authors:** Raquel A. Ortíz Cruz, José L. Cárdenas López, Gustavo A. González Aguilar, Humberto Astiazarán García, Shela Gorinstein, Rafael Canett Romero, Maribel Robles Sánchez

**Affiliations:** ^1^Departamento de Investigación y Posgrado en Alimentos, Universidad de Sonora, Boulevard Luis Encinas y Rosales, Colonia Centro, 83000 Hermosillo Sonora, SON, Mexico; ^2^Centro de Investigación en Alimentación y Desarrollo, A.C. (CIAD, A.C.), Carretera a La Victoria, Km 0.6, 83304 Hermosillo Sonora, SON, Mexico; ^3^Institute for Drug Research, School of Pharmacy, The Hebrew University of Jerusalem, P.O. Box 12065, 91120 Jerusalem, Israel

## Abstract

The aim of this study was to compare *in vitro* the antioxidant potential of sorghum kafirin and sorghum flour and their influence on lipids and antioxidant capacity in rats. The antioxidant activity in sorghum kafirin extract measured by the DPPH and TEAC methods was increased 30 and 65 times, respectively, compared to that of its counterpart, sorghum flour. According to electrophoresis assay, the kafirins *tert*-butanol extract showed a high proportion of *α*-kafirin monomers, and its amino acid composition revealed higher hydrophobic amino acid content such as alanine, isoleucine, leucine, tyrosine and phenylalanine than sorghum flour extract. Diets supplemented with sorghum kafirin extract have improved lipid metabolism and increased the serum antioxidant potential (67%) especially in rats fed with added cholesterol. The bioactive peptides generated from kafirin *in vivo* hydrolysis appear to be associated with the positive effect on serum lipids and antioxidant activity. According to these results, sorghum kafirin extract at the levels used in this study apparently could be used for prevention of atherosclerosis and other chronic diseases.

## 1. Introduction

Cardiovascular disease (CVD) is the most common cause of death in Mexico and atherosclerosis is the most prevalent CVD in the adult population, while coronary heart disease (CHD) is its most frequent and lethal form [1, 2]. The main risk factor of CVD is dyslipidemia characterized by elevated total cholesterol (TC), low density lipoprotein cholesterol (LDL-C) and triglyceride (TG) levels, and decreased high density lipoprotein cholesterol (HDL-C) levels [3]. Oxidative stress may play a critical role in the pathophysiology of CVD and several epidemiological studies have shown an association between circulating antioxidants and cardiovascular diseases [4–6]. Several investigations have found an association between whole grain intake and CVD reduction [7–9]. Phenolic compounds present in grains are responsible for this protecting effect, and also some proteins and their peptides have been recognized for their biological activity [10, 11].

*Sorghum* is studied because of its high content of fiber, protein, mineral, and polyphenol content, and several studies have shown its possible role in the cardiovascular disease prevention [12, 13].* In vitro* hydrolysis of sorghum protein has been studied for to its peptides and their relationship to antiviral [14] and antihypertensive activities [15]. These activities are attributed to fractions isolated from *α*-kafirin, the main storage protein in sorghum. The mechanisms by which kafirin can exert a protective effect start with hydrolysis and the absorption of biologically active peptides or amino acids, that can affect biological processes including the body function or health status [16].

Antioxidant activity is defined as the property that some chemicals have in quenching free radicals by donating a proton or hydrogen atom. Free radicals can oxidize biological structures such as lipids, which once oxidized can cause alterations in the cell membrane, while that their oxidation products can promote the development of atherogenic processes [17, 18]. It is possible that the enzymatic digestion of sorghum kafirins in an* in vivo *model may promote the production of bioactive peptides with antioxidant activity that can be absorbed and passed into the bloodstream, leading to a beneficial effect on its lipid profile. The aim of the study was to evaluate the* in vitro* potential antioxidant of sorghum kafirin extract as compared with sorghum flour and their effect on lipid profile and antioxidant activity in serum of hypercholesterolemic rats. The findings of this study are determinant in further understanding and development of this nonconventional cereal to be used in treatment of CVD.

## 2. Materials and Methods

### 2.1. Materials and Chemicals

*Sorghum* (*Sorghum bicolor *L. Moench) white variety UDG-110 was provided by Fundación Produce, Mexico. Cholesterol, 6-hydroxy-2,5,7,8-tetramethylchroman-2-carboxylic acid (Trolox), 2,2-azino-bis (3-ethylbenzthiazoline-6-sulfonic acid) (ABTS), 1,1-diphenyl-2-picrylhydrazyl (DPPH), fluorescein (FL), and 2,2′-azo-bis (2-amidinopropane) dihydrochloride (AAPH) were purchased from Sigma Chemical Co., St. Louis, MO, USA. Total cholesterol (TC), low density lipoprotein cholesterol (LDL-C), high density lipoprotein cholesterol (HDL-C), and triglyceride kits were acquired from RANDOX. Unless otherwise specified, all chemicals and solvents were of analytical grade. Sorghum flour (SF) (9.25% total protein dry matter basis) was obtained from whole grain using a laboratory mill (Laboratory Mill 1100), fitted with a 0.5 mm opening screen.

### 2.2. Extraction of Kafirins

The method of Mazhar et al. [19] was used for the extraction of kafirins. In brief, 100 g of defatted sorghum flour was mixed with 500 mL 60%* tert*-butanol/water at 37°C and vigorously stirred for 6 h. After centrifugation, the* tert*-butanol was evaporated to recover the supernatant. The aqueous supernatant containing kafirins was freeze-dried and named SK. The protein content of the SK was 40% (*N*  × 6.25, dry matter basis).

### 2.3. Sodium Dodecyl Sulfate Polyacrylamide Gel Electrophoresis

The SK was characterized by sodium dodecyl sulfate polyacrylamide gel electrophoresis procedure (SDS-PAGE) on 4–12% acrylamide gradient gel under reducing conditions [20, 21]. All gels were stained with Coomassie brilliant blue stain R-250 at 0.125%. The low MW markers (BIO-RAD) for gel electrophoresis were albumin (66 kDa), ovalbumin (45 kDa), glyceraldehyde 3-phosphate dehydrogenase (36 kDa), carbonic anhydrase (29 kDa), trypsinogen (24 kDa), trypsin inhibitor (20.1 kDa), and lactalbumin (14.4 kDa). Gels were analyzed with a GS-800 Bio Rad Densitometer using Quantity One version 4.6.9 software.

### 2.4. Amino Acid Composition Analysis

Amino acid analysis of samples of SF and SK was based on the methodology previously reported [22]. Briefly, powered samples (3 mg) were hydrolyzed with HCl (6 N) at 150°C during 12 hours. After hydrolysis, the acid was removed by rotary evaporation and the sample was resuspended on 2 mL of sodium citrate buffer pH 2.2. The HPLC method precision and accuracy was evaluated using external and internal standards. The amino acid reference standard consisted of sixteen amino acids (0.05 *μ*moles mL^−1^ each amino acid) and was utilized to determine the retention times for each amino acid. Internal standard *α*-aminobutyric (0.05 *μ*moles mL^−1^) was added to amino acid reference standard and each sample to normalize and quantify the amino acid content. A gradient mobile phase of sodium acetate 0.1 M pH 7.2 and methanol (9 : 1) elute sample for amino acid separation through C18 column reversed-phase octadecyl dimethylsilane particles (100 × 4.6 mm × 1/4′′ Microsorb 100-3 C18). Fluorescence detection was performed using an excitation emission wavelength of 360 and 455 nm, respectively. Star Chromatography work station (Varian version 5.51) software was used to achieve amino acid peak integration. The results (amount of amino acids g/100 g of protein) listed in Table 2 are means of three replications and coefficient of variation was lower than 5%.

### 2.5. Trolox Equivalent Antioxidant Capacity (TEAC)

This assay is based on the ability of antioxidants to scavenge the blue-green ABTS^•+^ radical cation, relative to the ABTS^•+^ scavenging ability of the water-soluble vitamin E analogue Trolox. The ABTS^•+^ radical cation was generated by the interaction of 5 mL of 7 mM ABTS solution and 88 *μ*L of 140 mM K_2_S_2_O_8_ solution. After the addition of 3.9 mL of ABTS^•+^ solution to 0.1 mL of methanolic (SF) or* tert*-butanol (SK) extracts or Trolox standards (0 to 20 *μ*M range), the absorbance was monitored exactly 1 and 30 min after the initial mixing. The percentage of absorbance inhibition at 734 nm was calculated and plotted as a function obtained for the extracts and the standard reference (Trolox). The final TEAC values were calculated by using a regression equation between the Trolox concentration and the inhibition percentage and expressed as millimol of Trolox equivalents per g of dry weight [23].

### 2.6. DPPH Assay

This assay is based on the measurement of the scavenging ability of antioxidants towards the stable radical DPPH relative to the DPPH scavenging ability of the water-soluble vitamin E analogue Trolox. Briefly, 3.9 mL aliquot of DPPH (0.0634 mM) solution was added to the test tubes and 0.1 mL of methanolic (SF) or* tert*-butanol (SK) extracts or Trolox standards (0 to 20 *μ*M range) was added and shaken vigorously. The tubes were allowed to stand at 27°C for 60 min. A control reaction was prepared as above without any extract, and methanol was used for the baseline correction. Changes in the absorbance of the samples were measured at 515 nm. Radical-scavenging activity was expressed as the inhibition percentage. The final DPPH values were calculated by using a regression equation between the Trolox concentration and the inhibition percentage and expressed as millimol of Trolox equivalents per g of dry weight [23].

### 2.7. Animals and Diets

All experimental procedures were approved on August 1, 2013, by the Ethics Committee of the Research Center in Food and Development (CIAD, A.C.), Hermosillo, Sonora, Mexico. The mean weight of Wistar rats (*n* = 30) used was 120 g and they were provided by the Experimental Animals Laboratory of Universidad de Sonora, Mexico. They were allowed to have free access to basal diet and tap water for 7 days before experiment. After one-week acclimatization, the rats were randomly divided into five groups (*n* = 6, each) and were given the five different dietary treatments. During 28 days period, two control groups were fed a basal diet (Con) or hypercholesterolemic diet (Chol) and the treatment groups were fed with the Chol diet plus 5% sorghum flour (Chol/SF5), Chol diet plus 10% sorghum flour (Chol/SF10), or Chol diet plus 0.25% sorghum kafirin (Chol/SK0.25). We used 0.25% SK in Chol diet with the objective that rats had a similar amount of kafirin to that of rats fed with the lowest sorghum flour added to diet (Chol/SF5). The calculation of this % was as follows: 100 g of SF contained 9.5% of total protein, where 60% is prolamin fraction (~5.7 g). From this amount, 80% are kafirins (~4.56 g) consequently; 5 g of sorghum flour has 0.25 g of kafirin.  Table 1 shows the percent composition of the diets for each experimental group.

The cholesterol batches were mixed carefully with the basal diet just before the diets were offered to the rats. All rats were fed* ad libitum* once a day at 10 a.m. and the intake of the diet and body weight were monitored weekly. All rats had unrestricted access to drinking water. At the conclusion of the experiment (day 28), all groups of rats were anesthetized using diethyl ether, and blood samples were taken from the left atrium of the heart. Serum was prepared for analysis including TC, HDL-C, LDL-C, and TG as described by RANDOX Labs.

### 2.8. Serum Antioxidant Activity Measured by Using Oxygen Radical Absorbance Capacity (ORAC) Assay

This assay measured the effect of antioxidant components of foods or biological fluids on the decline in FL fluorescence induced by AAPH, a peroxyl radical generator. The reaction mixture contained 1.7 mL of 75 mM phosphate buffer (pH 7), 100 *μ*L of 0.0102 mM FL, 100 *μ*L of 320 mM AAPH, and 100 *μ*L of each sample or several dilutions of the Trolox standard. FL, phosphate buffer, and samples were preincubated at 37°C for 15 min. The reaction was started by the addition of AAPH, and the fluorescence was measured and recorded every 5 min until the fluorescence of the last reading declined to <5% in respect to the initial reading (approximately 60 min). One blank and a maximum of 12 samples were analyzed at the same time. The excitation and emission wavelength was set as 484 and 515 nm, respectively. The final ORAC values were calculated by using a regression equation between the Trolox concentration and the net area under the FL decay curve and were expressed as millimol of Trolox equivalent per L [23]. The area under the curve (AUC) was calculated according to the following equation: 
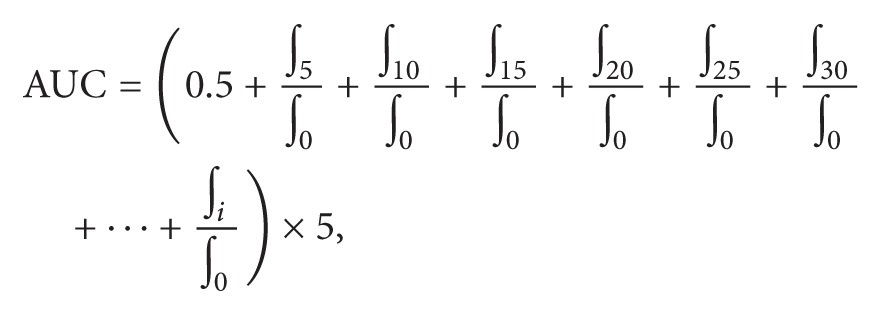
(1)where ∫_0_ is the initial fluorescence reading at 0 min and ∫_*i*_ is the fluorescence reading at time *i*. The data were analyzed by applying the equation in a Microsoft Excel spreadsheet to calculate AUC. The net AUC was obtained by subtracting the AUC of the blank from of the sample.

### 2.9. Statistical Analysis

The results of the* in vitro* study were analyzed by descriptive statistics, means ± SD, or coefficient of variance of three measurements. For the* in vivo* study, differences between groups were tested by two-way analysis of variance, followed by Tukey's multiple comparison test. The criterion for significance was *p* < 0.05.

## 3. Results and Discussion

### 3.1. Kafirin Extraction and Composition

**Figure 1:**
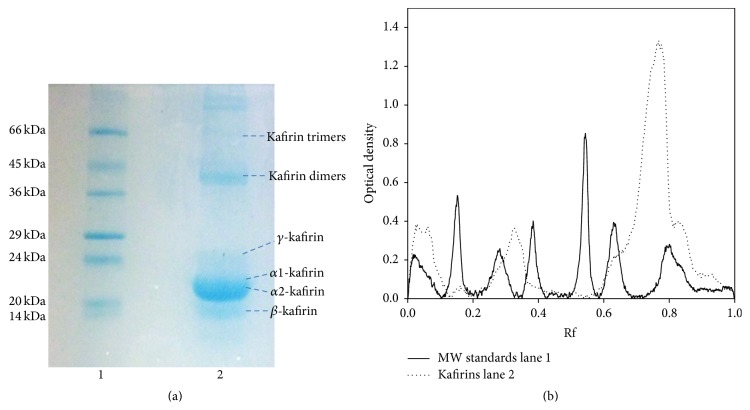
(a) SDS-PAGE pattern of kafirins from* Sorghum*. Lane 1: molecular weight standards; Lane 2: sorghum kafirin (SK) under reducing conditions. (b) Densitometry pattern of same 2 lanes.

Kafirins were extracted with 60%* tert*-butanol and analyzed under reducing conditions by SDS-PAGE.  Figure 1(a) shows the SDS PAGE pattern of kafirin from sorghum extract. Major bands were observed at 20–24 kDa, indicating the presence of *α*1- and *α*2-kafirin monomers. Bands close to 19 kDa and 27 kDa were identified as *β*-kafirin and *γ*-kafirin polypeptides, which corresponds with previous reports [24]. In addition, two bands close to 45 and 66 kDa were observed, suggesting the presence of dimers and trimers, respectively. These results were in agreement with the findings by Correia et al. [25] and Mehlo et al. [26] who used similar reducing conditions for the assay. Densitometry analysis showed that the *α*-kafirins had the highest optical density (OD) at 1.4 which is more than threefold of the other kafirins present in the extract (Figure 1(b)). Prolamins are storage proteins in sorghum grain and constitute 60% of the total protein. Several authors have reported that *α*-kafirins comprise 80–84% and *β*- and *γ*-kafirins constitute 7-8% and 9–12% of the total monomers, respectively [27].

Among the different options that make kafirin extraction more efficient is the use of a reducing agent in addition to* tert*-butanol. This induces a breakage of disulfide bonds between proteins and promotes the release of more kafirin monomers. However, the use of reducing agents such as 2-mercaptoethanol carries a high risk of toxicity; thus, given the goals of this study, this procedure is not considered appropriate.

Table 2 shows the amino acid composition of SF and SK samples. The amino acid composition of SF was similar to that reported by Mosse et al. [28], who studied the amino acid profile of 10* Sorghum* varieties. SK showed an amino acid composition different from that of SF, resulting in higher content of hydrophobic amino acids such as alanine, isoleucine, leucine, tyrosine, and phenylalanine. Our results agree with those reported by previous studies of kafirins obtained by similar extraction procedure [29]. Electrophoresis assay and the amino acid analysis of the sorghum kafirin extract were similar to those previously reported studies [27–29]. Therefore, protein obtained from* Sorghum* is referred to as kafirin. The extraction conditions were determinant on amino acid composition of kafirins. Nonreducing conditions and aqueous* tert*-butanol favored higher *α*-kafirin extraction rather than *β*- and *γ*-kafirin extraction. *α*-kafirins are monomers with a high affinity to aqueous* tert*-butanol and are characterized by high hydrophobic amino acid content but low proline and methionine content [27, 30–32].

It has been reported that the presence of hydrophobic amino acids is an indicator of antioxidant activity in several cereals or food plants extracts [33–35]. These authors reported that hydrophobic amino acids can inhibit lipid oxidation by hydrogen transference or metal chelation. However, other studies have documented that antioxidant activity increases with peptide sequences when the amino acids proline, leucine, and histidine are the most abundant [36, 37].

In our study, we observed that the efficacy of the sorghum flour to scavenge DPPH and ABTS radicals was lower than that of sorghum kafirin (Table 3). Antioxidant activity in sorghum flour can be attributed mainly to the presence of phenolic compounds, which are located in different proportions in the endosperm and pericarp. However, we cannot discriminate the possible contribution of some proteins and fatty acids present in sorghum flour to the antioxidant activity.

In white* Sorghum*, Awika et al. [38] reported lower antioxidant activity values (6 *μ*molTE/g) in DPPH and TEAC assays. To our knowledge, there are no previous studies of the antioxidant activity of sorghum kafirin extract. Therefore, new information about the antioxidant properties of these proteins is being reported. Regarding the antioxidant activity of SK extract, this could be attributed essentially to the presence of hydrophobic amino acids, as seen above.

### 3.2. Lipid Profile and Antioxidant Evaluation

**Figure 2:**
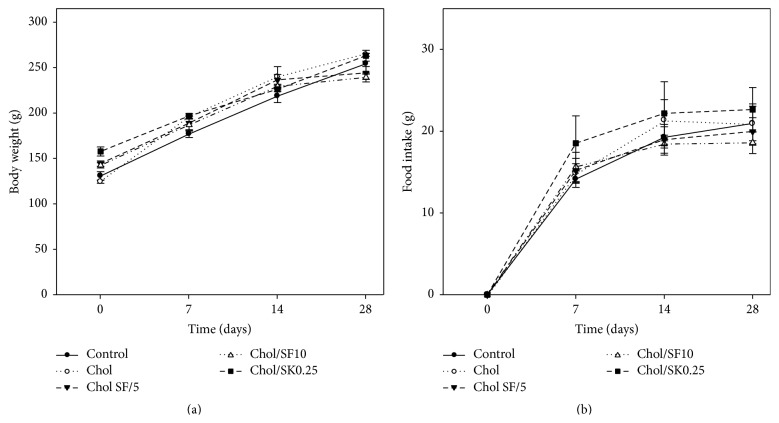
Body weight (a) and food intake (b) changes over the study period for each group of rats. Each value is the mean ± SD (*n* = 6) of each dietary group.

The data on body weight and food intake are shown in Figure 2.  Figure 2(a) shows the overview of changes in body weight over the study period for the different rat groups; no significant differences in weight between the different groups were observed.  Figure 2(b) shows that food intake was similar (*p* > 0.05) for each group. These observations indicate that the diet used in this study was well tolerated by rats.

SK supplemented diet in cholesterol fed groups after 4 weeks of feeding (Table 4) significantly hindered the rise of TC (1.74 ± 0.19 versus 2.12 ± 0.67 mmol/L) and LDL-C (0.84 ± 0.14 versus 1.33 ± 0.81 mmol/L). The same diets significantly increased HDL-C levels (0.96 ± 0.12 versus 0.68 ± 0.32 mmol/L).

A different behavior was observed in SF supplemented diets in the cholesterol fed groups after 4 weeks of feeding. The mean values of TC increased in both SF5% and SF10% (2.12 ± 0.67 to 2.75 ± 0.26 mmol/L, +29% and 2.12 ± 0.67 to 2.67 ± 0.26 mmol/L, and +26%, resp.). However, for the same diets, there was a significant increase in HDL-C content (0.68 ± 0.32 to 1.50 ± 0.17 and 1.36 ± 0.31, resp.).

**Figure 3:**
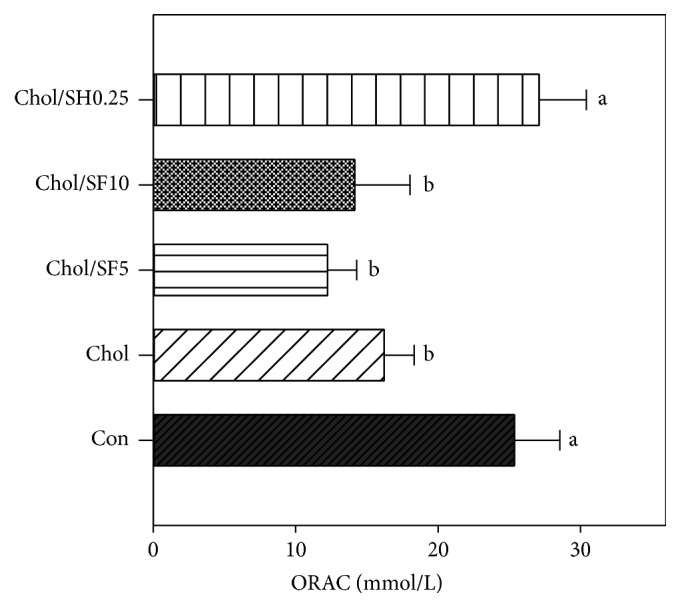
Oxygen Radical Absorption Capacity (ORAC) in rats fed with cholesterol. Each value is the mean ± SD (*n* = 6) of each dietary group. Bars with different letters are significantly different (*p* < 0.05).

At the end of the experiment, a significant increase in the serum antioxidant activity in the Chol/SK0.25 dietary group was observed with respect to Chol dietary group (16.22 ± 3.19 versus 27.10 ± 3.32 mmol/L). However, a decrease (*p* > 0.05) in the serum antioxidant activity after completion of the trial was registered in SF (5 and 10%) groups, with respect to Chol group diet (Figure 3).

This investigation has shown that sorghum kafirin positively influences the serum antioxidant activity in rats fed with added cholesterol. As far as we know, this is the first study that evaluates the* in vivo* changes in the lipid profile of the kafirin fractions of* Sorghum*. The mechanisms involved may explain that this favorable effect may be very different, ranging from a reduction in intestinal absorption of cholesterol and/or bile acids, a decrease in serum cholesterol favored by the activity of LDL receptors in the liver, and changes in the biotransformation of liver cholesterol [39].

With the results obtained in this study, we considered that kafirins could be partially hydrolyzed by digestive enzymes in the rat generating peptides that apparently are absorbed in the intestine resulting in different metabolic effects, especially in cholesterol metabolism.

Studies have shown that protein intake reduces cholesterol levels in circulation, and the mechanisms which have been attributed to this effect may be related to a reduction in total cholesterol synthesis [40]. Apparently, the bioactive peptides produced by an incomplete digestion of the protein affect cholesterol absorption in the digestive tract or have a direct effect on cholesterol synthesis and/or LDL receptor activity (LDLR) [41, 42].

Cereal grains are one of the most important sources of protein, and the storage proteins of these grains (prolamins) contain bioactive fragments. The bioactivity of these proteins has been extensively reviewed and it has been reported that prolamins are potential precursors of antihypertensive peptides [15]. Also, it has been found that the antioxidant activity of some peptides derived from whey or grains such as wheat may be attributed principally to hydrophobic amino acids such as leucine, proline, and also histidine [43, 44]. In the case of kafirin supplemented diets with cholesterol added, the antioxidant activity was kept at constant levels in circulation and even slightly higher than those achieved by the control group. This increase in serum antioxidant capacity may be related to the* in vivo* dietary ingested kafirin digestion, thus generating bioactive peptides with antioxidant activity that may be able to maintain serum levels of total cholesterol.

Another interesting result obtained in our study was the increase in TC values in the sorghum flour (5 and 10%) dietary groups fed with cholesterol, compared to the cholesterol group. Several authors have reported that this behavior had a positive influence on the inhibition of atherosclerosis [45, 46]. The mechanisms that could explain this are related to the inhibition of CETP (cholesteryl ester transfer protein), which is responsible for cholesterol esters of HDL transfer to other cholesterol fractions. When CETP is inhibited, there is accumulation of HDL-C, which has been recognized as antiatherogenic [47].

## 4. Conclusion

Our results demonstrated that sorghum kafirin extract had a good antioxidant potential in both* in vitro* and* in vivo* studies. The densitometric assay from electrophoresis pattern of kafirin fraction of sorghum flour showed that higher *α*-kafirins proportion in respect to other kafirins was found. Therefore, *α*-kafirins could be responsible of the antioxidant activity observed* in vitro*. The* in vivo* study confirmed that sorghum kafirin reduced the TC levels and increased the HDL-C levels in hyperlipidemic rats, suggesting that sorghum kafirin fraction can potentially reduce the risk of CVD. We concluded that sorghum flour consumption at the levels studied apparently protect against an atherosclerotic event.

## Figures and Tables

**Table 1:** Percent composition of experimental diets.

Components	Con	Chol	Chol/ SF5	Chol/ SF10	Chol/ SK0.25
Corn starch	69.1	68.1	63.1	58.1	67.85
Casein	15	15	15	15	15
Soybean oil	10	10	10	10	10
Cellulose	1	1	1	1	1
Mineral mixture	3.7	3.7	3.7	3.7	3.7
Vitamin mixture	1	1	1	1	1
Choline	0.2	0.2	0.2	0.2	0.2
Cholesterol	—	1	1	1	1
Sorghum flour (SF)	—	—	5	10	—
Sorghum kafirin (SK)	—	—	—	—	0.25

Con: control diet group; Chol: cholesterol diet group; Chol/SF5: 5% sorghum flour diet group; Chol/SF10: 10% sorghum flour; Chol/SK: 0.25% sorghum kafirin diet group.

**Table 2:** Amino acid composition of sorghum flour (SF) and sorghum kafirin (SK)^1^.

Amino acid	SF	SK
	g/100 g dry weight	
Asparagine	5.81	6.68
Threonine	2.92	2.32
Serine	3.25	3.29
Glutamic acid	14.84	23.12
Proline	7.21	3.03
Glycine	6.32	3.20
Alanine	9.24	14.08
Valine	4.83	4.29
Methionine	1.31	0.746
Isoleucine	3.92	4.01
Leucine	10.10	14.17
Tyrosine	5.31	5.51
Phenylalanine	5.05	5.79
Histidine	2.22	1.19
Lysine	3.06	1.01
Arginine	5.70	3.16

^1^Each value is the mean of three replications. The coefficient of variation was lower than 5%.

**Table 3:** Antioxidant activity in sorghum flour (SF) and sorghum kafirin (SK)^1^.

Sample	DPPH	TEAC
	mmol TE/g dry weight	
SF	0.101 ± 0.001^b^	2.74 ± 0.04^b^
SK	3.03 ± 0.37^a^	181.54 ± 1.76^a^

^1^Each value is the mean ± SD of three replications. Means in columns not followed by common letters differ significantly (*p* < 0.05).

**Table 4:** Changes in the serum lipid profile of rats fed with different diets: 1% cholesterol (Chol), 5% and 10% sorghum flour, and 0.25% sorghum kafirin^1^.

Dietary group	TC	HDL-C	LDL-C	TG
	mmol/L			
Control	1.46 ± 0.10^d^	0.71 ± 0.06^c^	0.75 ± 0.06^b^	0.99 ± 0.18^a^
Chol	2.12 ± 0.67^b^	0.68 ± 0.32^c^	1.33 ± 0.81^a^	0.75 ± 0.32^ab^
Chol/SF5	2.75 ± 0.26^a^	1.50 ± 0.17^a^	1.17 ± 0.16^a^	0.80 ± 0.09^ab^
Chol/SF10	2.67 ± 0.26^a^	1.36 ± 0.31^a^	1.23 ± 0.21^a^	0.67 ± 0.07^b^
Chol/SK0.25	1.74 ± 0.19^c^	0.96 ± 0.12^b^	0.84 ± 0.14^b^	0.61 ± 0.09^b^

^1^Each value is the mean ± SD (*n* = 6) of each dietary group. Means in columns not followed by common letters differ significantly (*p* < 0.05).

TC: total cholesterol; HDL-C: HDL cholesterol; LDL-C: LDL cholesterol; TG: Triglycerides; Chol: nonoxidized cholesterol; SF: sorghum flour; SG: sorghum kafirin.
